# VLPs containing stalk domain and ectodomain of matrix protein 2 of influenza induce protection in mice

**DOI:** 10.1186/s12985-023-01994-4

**Published:** 2023-02-27

**Authors:** Lili Shi, Ying Long, Yanyan Zhu, Jingjian Dong, Yan Chen, Hao Feng, Xianliang Sun

**Affiliations:** 1 Medical School of Jiaxing University, Jiahang Road 118, Nanhu District, Jiaxing, 314001 Zhejiang People’s Republic of China; 2 School of Medicine, and The First Affiliated Hospital, Huzhou University, 759 2Nd Ring East Road, Huzhou, 313000 Zhejiang People’s Republic of China; 3 Zhejiang Chinese Medical University (Jiaxing University Master Degree Cultivation Base), Bin Wen Road 548, Binjiang District, Hangzhou, 310053 Zhejiang People’s Republic of China

**Keywords:** Influenza viruses, Stalk domain, M2e, Virus-like particles, Cross-protection

## Abstract

**Background:**

As a result of antigenic drift, current influenza vaccines provide limited protection against circulating influenza viruses, and vaccines with broad cross protection are urgently needed. Hemagglutinin stalk domain and ectodomain of matrix protein 2 are highly conserved among influenza viruses and have great potential for use as a universal vaccine.

**Methods:**

In this study, we co-expressed the stalk domain and M2e on the surface of cell membranes and generated chimeric and standard virus-like particles of influenza to improve antigen immunogenicity. We subsequently immunized BALB/c mice through intranasal and intramuscular routes.

**Results:**

Data obtained demonstrated that vaccination with VLPs elicited high levels of serum-specific IgG (approximately 30-fold higher than that obtained with soluble protein), induced increased ADCC activity to the influenza virus, and enhanced T cell as well as mucosal immune responses. Furthermore, mice immunized by VLP had elevated level of mucosal HA and 4M2e specific IgA titers and cytokine production as compared to mice immunized with soluble protein. Additionally, the VLP-immunized group exhibited long-lasting humoral antibody responses and effectively reduced lung viral titers after the challenge. Compared to the 4M2e-VLP and mHA-VLP groups, the chimeric VLP group experienced cross-protection against the lethal challenge with homologous and heterologous viruses. The stalk domain specific antibody conferred better protection than the 4M2e specific antibody.

**Conclusion:**

Our findings demonstrated that the chimeric VLPs anchored with the stalk domain and M2e showed efficacy in reducing viral loads after the influenza virus challenge in the mice model. This antibody can be used in humans to broadly protect against a variety of influenza virus subtypes. The chimeric VLPs represent a novel approach to increase antigen immunogenicity and are promising candidates for a universal influenza vaccine.

## Introduction

Influenza virus infection is a major public health problem worldwide, and seasonal epidemics account for 3–5 million cases of severe illness and nearly 290,000–650,000 deaths globally each year [1]. Vaccination against infectious diseases is one of the most cost-effective strategies. Current licensed influenza vaccines are mainly based on hemagglutinin (HA) antigen, which is highly variable among different influenza viruses. The immune responses induced by these vaccines provide limited protection and the circulating viral strains vary because of the antigenic drift driven by adaptive immune response in the population [2, 3]. Such vaccines elicit neutralizing antibodies directed to the antigenic sites in the variable globular head domain of HA. The antibodies are generally strain specific, thereby causing a decrease in vaccine efficacy [4, 5]. Thus, seasonal influenza vaccines need to be updated annually [6].

Many studies have been conducted to develop universal vaccines with broader and longer-lasting protection. Universal vaccines have focused on highly conserved regions among different virus subtypes, including the ectodomain of matrix 2 protein (M2e) and the HA stalk domain, which have been extensively investigated as a promising antigenic target for developing a more effective universal vaccine. M2 protein is a transmembrane, homotetrameric proton ion channel involved in virus uncoating following entry [7]. The N-terminal 9 amino acids in M2e have been found to be conserved at 100% in the human influenza A virus [8], and the sequence homology of the stalk domain among various subtypes is in the range of 51–80%. The stalk domain and M2e are relatively well conserved but far less immunogenic, and under normal conditions, antibodies against these domains seem rare and too weak to afford protection [9]. Various approaches have shown promise in the development of universal vaccines mainly focused on stalk domain and M2e. For example, Steel et al. [10] designed a headless construct that was more effective than controls in protecting immunized mice from homologous challenge. Palese et al. [11] constructed chimeric HA molecules and induced antibodies against the stalk domain to protect animals from homologous and heterologous virus challenges [11]. Many studies have shown that this highly conserved region represents a vaccine strategy that may aid in pandemic preparedness. Because the M2e sequence is highly conserved across the influenza virus, it is also a promising antigenic target for developing a universal vaccine [12, 13].

Our previous study [14] showed that virus-like particles (VLPs) incorporating the stalk domain promoted effective immune responses and exhibited high titers of ADCC antibodies. In the present study, the stalk domain and M2e were co-anchored to the surface particles and generated chimeric influenza VLPs. The immunogenicity and protection of the generated cVLPs were tested in mice.

## Materials and methods

### Ethics statement

This study was approved by the Animal Care and Use Committee of Jiaxing University. All animal experiments were performed in accordance with the committee’s guidelines. Anesthesia was administered before immunization and sampling.

### Cell lines and viruses

Sf9 insect cells were maintained in SF900II (Life Technologies, San Diego, CA, USA) at 27 °C in cell culture. Influenza virus A/Aichi/68 H3N2 and A/Anhui/2013 H7N9 provided by Dr. Zhu Hongwei (Ludong University) were propagated in the allantoic cavities of 10-day-old embryonated hen’s eggs at 37 °C for 2 d. Allantoic fluid was harvested and centrifuged at 2000 rpm for 10 min. The viruses were titrated by infection of mice with serial dilutions, and LD_50_ (50% lethal dose) was calculated using the method of Reed and Muench.

### Expression and characterization of influenza VLPs

The HA, M2e, and M1 genes were from the H3N2 sequence. The stalk domain (mHA) construction was based on our previous reports, the head domain of HA was replaced by peptide linker (GGGGGS)4 [14], and the M2e amino acid sequence is MSLLTEVETPIRNEWGCRCND. The coding sequence of glycosylphosphatidyl-inositol-GPI anchor was fused to the ends of the mHA and 4M2e coding gene to generate the gene encoding the membrane-anchored mHA and four individual repeat M2e (4M2e). The full-length construct encoding a GPI-anchored mHA and 4M2e was confirmed by DNA sequencing. The gene encoding M1, membrane anchored mHA, and M2e protein were cloned into pFastBac1. Recombinant baculovirus (rBVs) expressing mHA, 4M2e, and M1 were generated by using a Bac-to-Bac expression system (Invitrogen, Carlsbad, CA) according to the manufacturer’s protocol. Three different VLPs, namely, (mHA + 4M2e) VLP (chimeric VLPs, cVLPs) and standard VLP group (mHA VLP and 4M2e VLP), were produced by an insect cell expression system. For cVLPs, three rBVs expressing influenza M1, GPI-4M2e, and GPI-mHA co-infected sf9 cells at multiplicity of infection (MOI) of 1:4:2. Then, 4M2e VLP was produced by co-infection of sf9 cells with rBVs expressing 4M2e and M1 at MOI of 2:1. mHA VLP were produced by co-infection of sf9 cells with rBVs expressing mHA and M1 at MOI of 2:1. After 48–72 h infection, the culture supernatant was collected and VLPs were concentrated by experimental tangential flow concentration and purification dialysis system (Merck Millipore, Germany) followed by sucrose density gradient ultracentrifugation. The VLPs were characterized by Western blotting using antibodies against HA and M2e (R&D system). VLP protein concentration was determined by ELISA in which purified proteins were used to generate the quantitative standard curve. Bio-Rad protein assay (Bio-Rad Laboratories Inc., Hercules, USA) was used to quantify the yield of mHA and M2e protein in VLP.

### Immunization, sample collection, and challenge

**Fig. 1:**
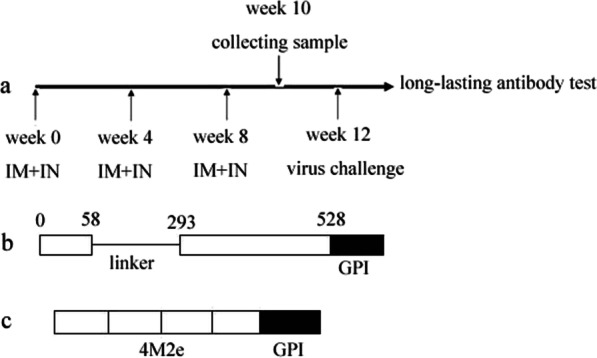
Immunization schedule and fusion gene constructs. **a**, immunization schedule, the mice were immunized three times at four weeks intervals through IM and IN routes, samples were collected at two weeks after final immunization, mice were challenged at four weeks after final immunization, partial mice were kept for the long-lasting antibody test in each group. **b**, **c** diagrams of 4M2e and HA stalk domain fusion gene. **b**, to generate constructs HA stalk domain, the sequence corresponding HA globular head domain (amino acid 59–292) were replaced by a flexible linker sequence encoding (GGGGGS)4. **c**, 4M2e, four M2e copies in tandem repeat form. The GPI anchor was fused to the ends of the HA stalk domain and 4M2e coding gene

Three- to four-week-old female BALB/c mice were separated into five groups, 15 mice for each group. The mice were immunized with soluble mHA, 4M2e, or influenza VLPs through intramuscular (IM) and intranasal (IN) administration, followed by two boosts at four-week intervals. Group 1 (G1) mice were immunized with soluble mHA protein. Group 2 (G2) mice were immunized with soluble 4M2e protein, Group 3 (G3) mice were given 4M2e-VLP. Group 4 (G4) mice received mHA-VLP. Group 5 (G5) mice were immunized with cVLPs. The mice were immunized with 50 μg target protein everytime and 25 μg for each administration route. Samples were collected two weeks after final immunization. Blood samples were collected by retro-orbital plexus puncture, and nasal washes were collected by lavaging the mice nostrils repetitively with 200 μl PBS containing 0.05% Tween 20 (PBST). Supernatants were collected after centrifugation (5,000 rpm for 10 min). Serum and mucosal samples were stored at − 80 °C for further assays. Lymphocytes from spleen samples collected from mice sacrificed two weeks after the final boost were used for cytokine assays. Four weeks after the final boost, the mice were challenged with 5 × LD_50_ of mouse-adapted influenza virus. Body weight loss and survival rates were monitored daily for 14 d post infection, Fig. 1a.

### Antibody levels and lung virus loads

mHA and 4M2e-specific antibody titers in serum were measured by ELISA. For mHA -specific antibody test, soluble mHA was used as coating antigens. For M2e-specific antibody test, soluble 4M2e was used as coating antigens. ELISA plates were coated with soluble protein (250 ng/well) overnight, plates were washed three times with PBS plus 0.05% Tween-20 and blocked with 1% BSA in PBS for 1 h at 37 °C. Following washes, serial dilutions of samples were added to the plate and incubated for 1 h at 37 °C. Second antibody, goat HRP-conjugated anti-mouse IgG antibody was added and incubated for 1 h at 37 °C. After wash, the tetramethylbenzidine (TMB, R&D Systems, USA) was added and the OD value was read in an ELISA plate reader using a test wavelength of 450 nm. The highest dilution factor that gives an OD 450 of twice that of the naive sample at the dilution was designated as the antibody end point titer. For the lung virus titers, the mouse lungs were collected in each group 4 d post challenge and ground into lung homogenates. Then, they were centrifuged at 1000 rpm for 10 min to remove tissue debris. The lung virus titers were determined by MDCK cell-based plaque assay as described by Wang et al. [15]. For the long-lasting antibody test, three mice in each group were kept without virus challenge. Samples were collected one, three, and six months after final immunization, Fig. 1.

### ADCC (antibody-dependent cell-mediated cytotoxicity, ADCC) assay

The chromium-release assay were used to calculate the percentage specific immune lysis (SIL) of infected A549 cells [16, 17]. A549 cells were transfected with plasmid DNA encoding for HA stalk domain or M2 of H3N2 and H7N9 using Lipofectamine2000 (Invitrogen) two days before the experiment, for chimeric VLPs group, A549 cells were co-transfected with plasmid encoding HA stalk domain and M2. One day before the assay, transfected cells were harvested and washed, centrifuged, and labeled with ^51^Chromium for 1 h, the labeled cells were washed and added at 2000 cells/well in 96-well plates, fourfold serial dilution of heat-inactivated serum were added to wells in replicates of three, then plates were incubated at 37℃. NK cells were purified through MagnisortTM Mouse NK cell Enrichment Kit (Fisher Scientific Inc., Rockford, IL), enriched NK cells were added at an E:T (effector:target) ratio of 5:1/well. Plates were spun at 250 × g for 5 min and thenincubated at 37℃ in a culture incubator for 2 h. Supernatants were harvested and counted in a gamma counter. Calculation of %SIL at each serum dilution = %lysis of sample– %lysis of NK cells alone. Calculation of %lysis at each serum dilution = (average CPM of experimental release – average CPM ofminimum release)/(average CPM of maximum release – average CPM of minimum release) × 100. Calculation of %lysis of NK cells = (average CPM of NK cell release—average CPM of minimum release)/(average CPM of maximum release—average CPM of minimum release) × 100. CPM (count per minute), lysis of transfected A549 cells by NK cells alone without sera was against transfected cells, when calculating SIL values, data on lysis of NK cells alone were subtracted. The highest serum dilution showing ≥ 15% SIL was defined as ADCC antibody end point titer. For calculation of mean endpoint titers, an undetectable ADCC titer was assigned a value of 5.

### Cytokine assays

Interferon gamma (INF-γ) and interleukin 4 (IL-4) secretions from immunized mouse splenocytes were evaluated using ELISA kits (R&D System, Minneapolis, USA) in accordance with the manufacturer’s instructions.

### Statistical analysis

The analyses were performed by using GraphPad Prism version 5.00 for Windows (GraphPad Software, San Diego, CA). P values less than 0.05 (*p* < 0.05) were considered statistically significant. **p < 0.01, n.s.,*p* > 0.05.

## Results

### Preparation and characterization of VLPs

**Fig. 2:**
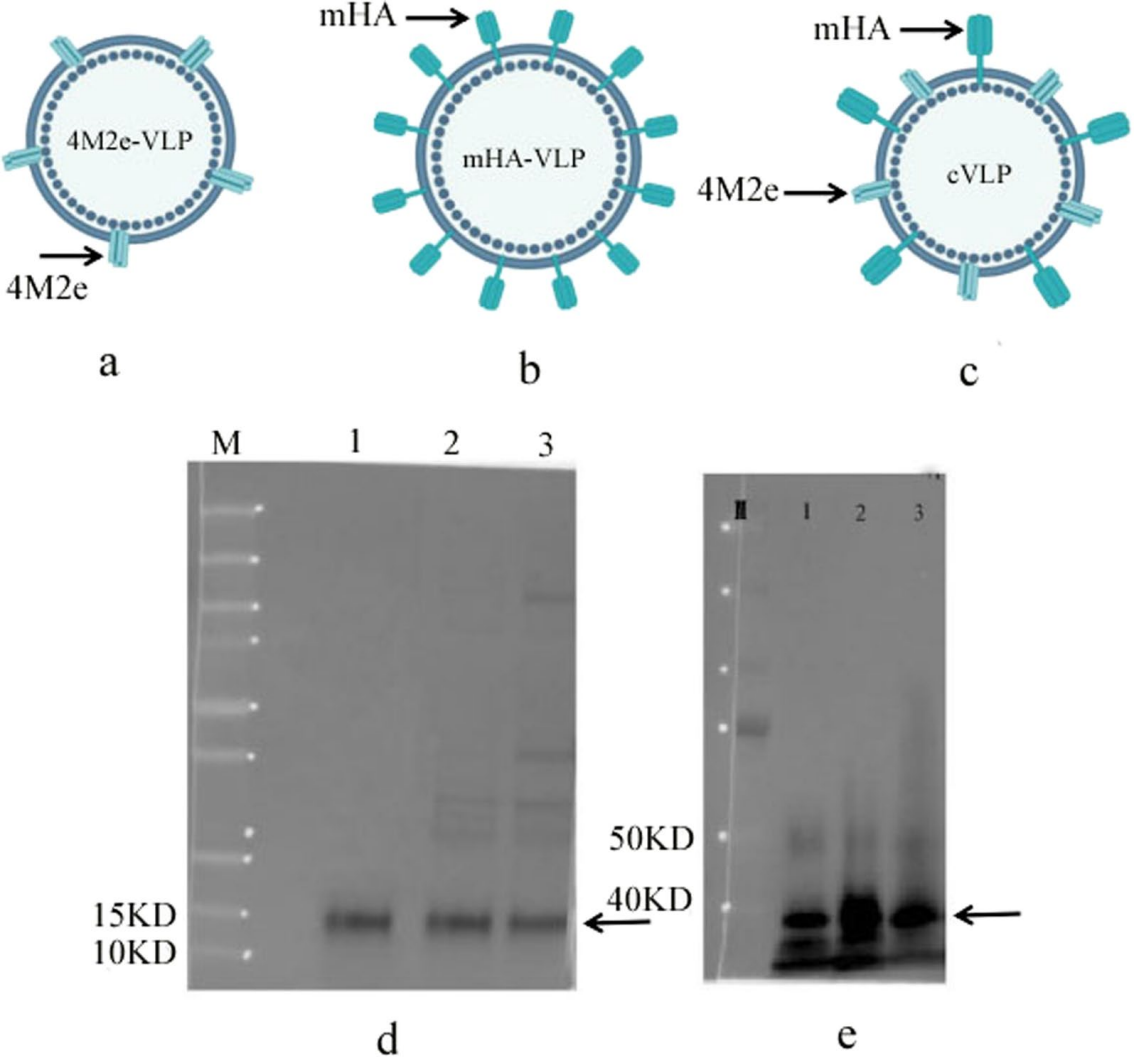
Schematic diagram and characterization of influenza VLPs. **a**–**c**, VLPs schematics, the VLPs were prepared as described in materials and methods. **a**, 4M2e-VLP, 4M2e anchored on the surface of particles. **b**, mHA-VLP, mHA anchored on the surface of particles. **c**, chimeric VLP, 4M2e and mHA co-anchored on the surface of particles. Characterization of VLPs, **d**, western blotting analysis of 4M2e protein. M, molecular weight (kD). Lane 1, 4M2e soluble protein. Lane 2, 4M2e-VLPs. Lane 3, cVLPs. e, western blotting analysis of mHA protein. M, molecular weight (kD). Lane 1, mHA soluble protein. Lane 2, mHA-VLPs. Lane 3, cVLPs. The mouse anti HA and M2 primary antibody were diluted at 1:500, secondary antibody were diluted at 1:2500

The diagrams of 4M2e and HA stalk domain fusion gene constructs are as shown in Fig. 1b, c. VLP constructs were designed as shown in Fig. 2a–c, The VLPs were purified by sucrose density gradient ultracentrifugation. 4M2e-VLP was observed to have a molecular mass of approximately 13 kDa as shown in Fig. 2d. mHA-VLP was observed to have a molecular mass of approximately 40 kDa as shown in Fig. 2e. cVLP was observed to have two bands with molecular mass of 40 kDa and 13 kDa, indicated by arrow.

### VLPs induced strong humoral and mucosal antibody responses

**Fig. 3:**
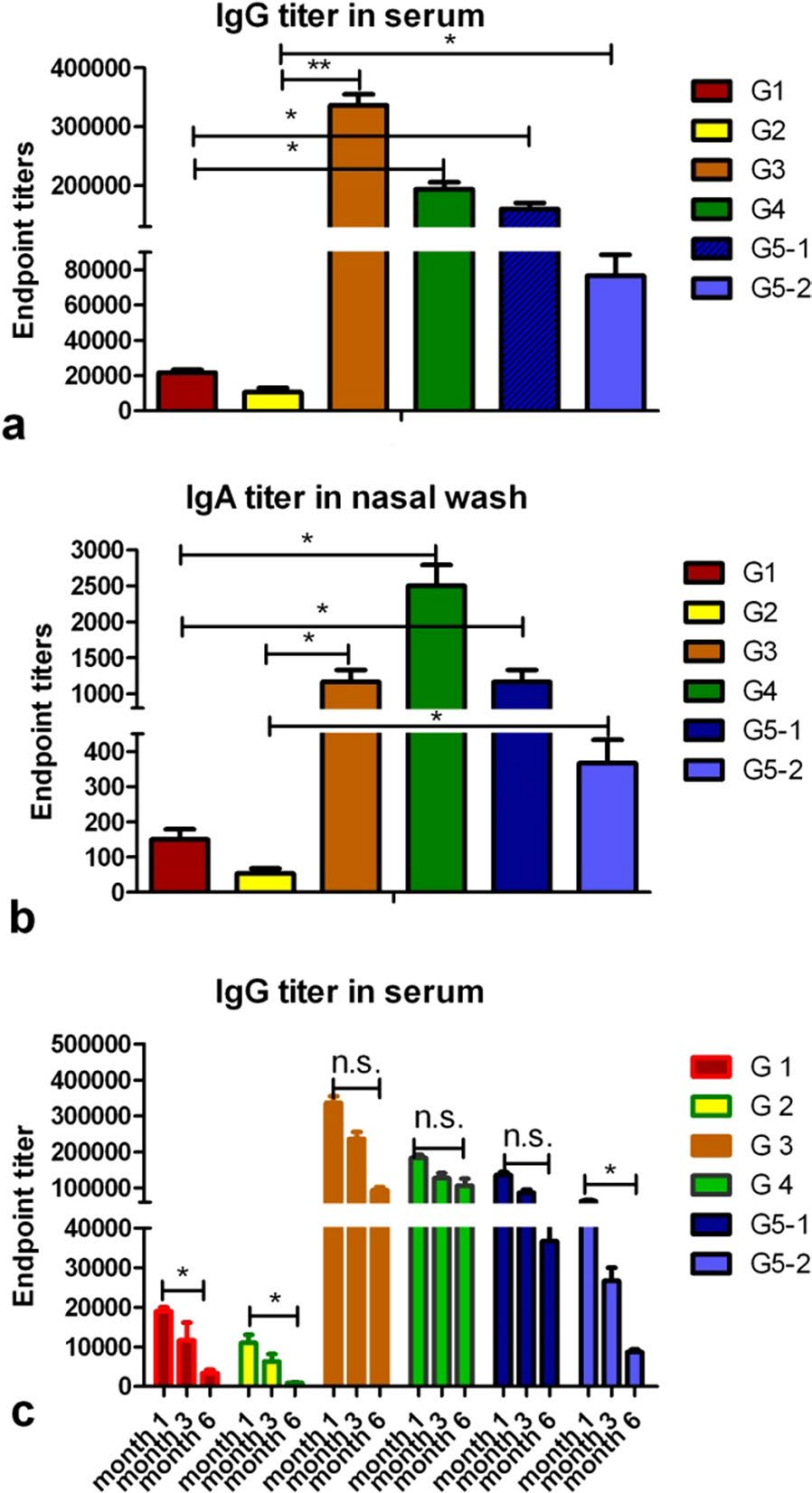
Antibody responses. Mice were immunized with soluble protein and VLPs through intranasal and intramuscular routes. The serum and nasal washes sample were collected 2 weeks after the final immunization. **a**, serum IgG titers. **b**, IgA titers in nasal washes. **c**, long lasting antibody responses were measured in mice sera at time points of 1, 3 and 6 months after final immunization. Results are expressed as means ± standard deviations. *P < 0.05 were considered statistically significant

The immune sera and mucosal samples were evaluated for antigen-specific IgG and IgA titers using ELISA with soluble 4M2e and mHA as coating antigens. 4M2e-specific IgG titers in sera of 4M2e-VLP immunized group were approximately 30-fold higher than those of soluble 4M2e protein immunized group (p < 0.01) as demonstrated in Fig. 3a. mHA-specific IgG titers in sera of mHA-VLP immunized group were approximately tenfold higher than those of soluble mHA protein immunized group (p < 0.05) as shown in Fig. 3a. In the cVLP immunized groups, 4M2e-specific IgG titers(G5-2) in sera were approximately seven-fold higher than those of soluble 4M2e protein immunized group (p < 0.05). The mHA-specific IgG titers (G5-1) in sera were approximately seven- to eight-fold higher than that of soluble mHA protein immunized group (p < 0.05). In cVLP immunized group, 4M2e and mHA-specific IgG titers were lower than those of the 4M2e-VLP and mHA-VLP immunized groups.

Figure 3b shows that the VLP immunization induced enhanced mucosal responses as compared to the soluble protein immunization. mHA-specific IgA end-point titers in nasal washes from the mHA-VLP group were six fold higher than those from the mice immunized by soluble mHA protein. 4M2e-specific IgA end-point titers in nasal washes from the 4M2e-VLP group were six fold higher than those of mice immunized by soluble 4M2e protein. 4M2e and mHA-specific IgA titers were similar between the cVLP and standard VLP groups.

Furthermore, we determined whether VLP vaccination could induce long-lasting protective antibody responses. As shown in Fig. 3c, in 4M2e-VLP and mHA-VLP immunized groups, the IgG titers were maintained at comparable levels after three months, while an approximate 50% decrease in IgG titers was observed (P > 0.05) after six months. By contrast, the IgG titers dropped after three months in the two soluble protein immunization groups and the antibody titers decreased significantly after six months (P < 0.05). The results demonstrated that the VLPs induced long-lasting protective humoral immune responses in mice.

### ADCC responses

**Fig. 4:**
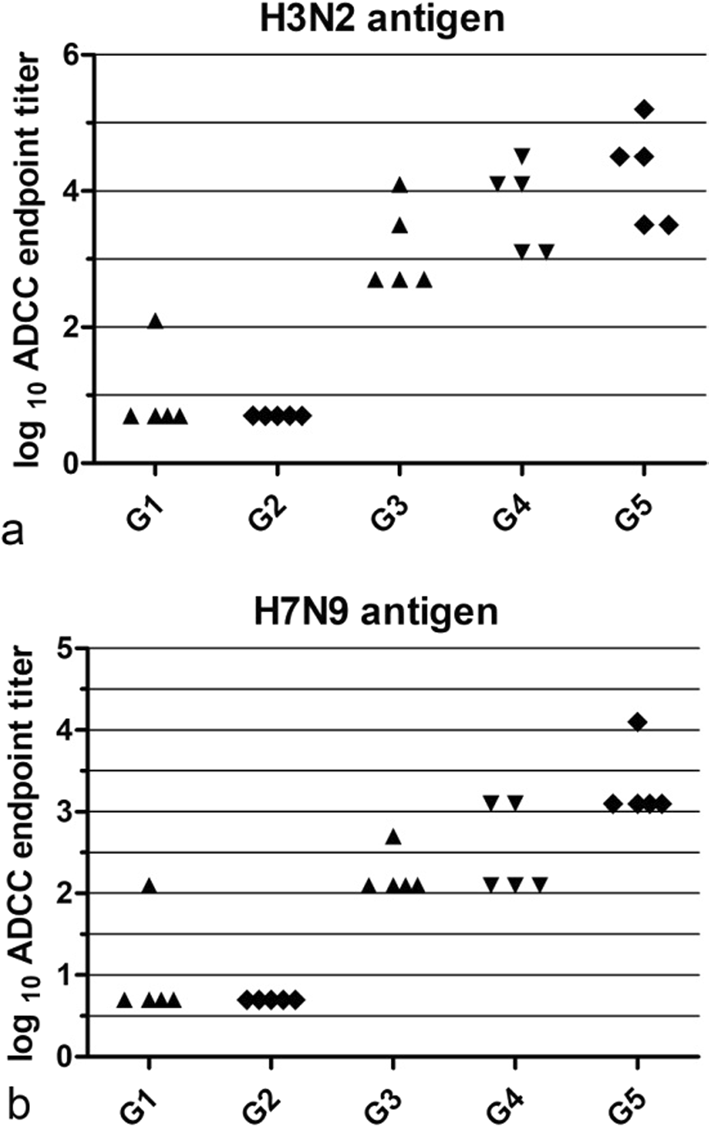
ADCC assay. ADCC antibody titers in VLP and soluble protein immunized group, the panels show log10 ADCC antibody titers, the lowest dilution tested was 1:20(fourfold serial dilution were used). Samples under the detection limit were plotted at 0.7 in the log10 scale(equivalent to 1:5 dilution). The experiment was performed in triplicate. **a**, H3N2 antigen group. **b**, H7N9 antigen group

The evaluation of ADCC responses demonstrated that sera from 1 out of 5 mice in soluble mHA group and all mice in VLPs group had detectable ADCC antibodies. Compared to soluble immunized group, sera from VLPs immunized group exhibited increased ADCC activity in H3N2 antigen group, fig. For H7N9 antigen group, 1 out of 5 mice had detectable ADCC antibodies in soluble mHA group, compared to soluble immunized group, sera from VLPs immunized group got increased ADCC activity, shown in Fig. 4.

### VLPs stimulate T-cell responses

**Fig. 5:**
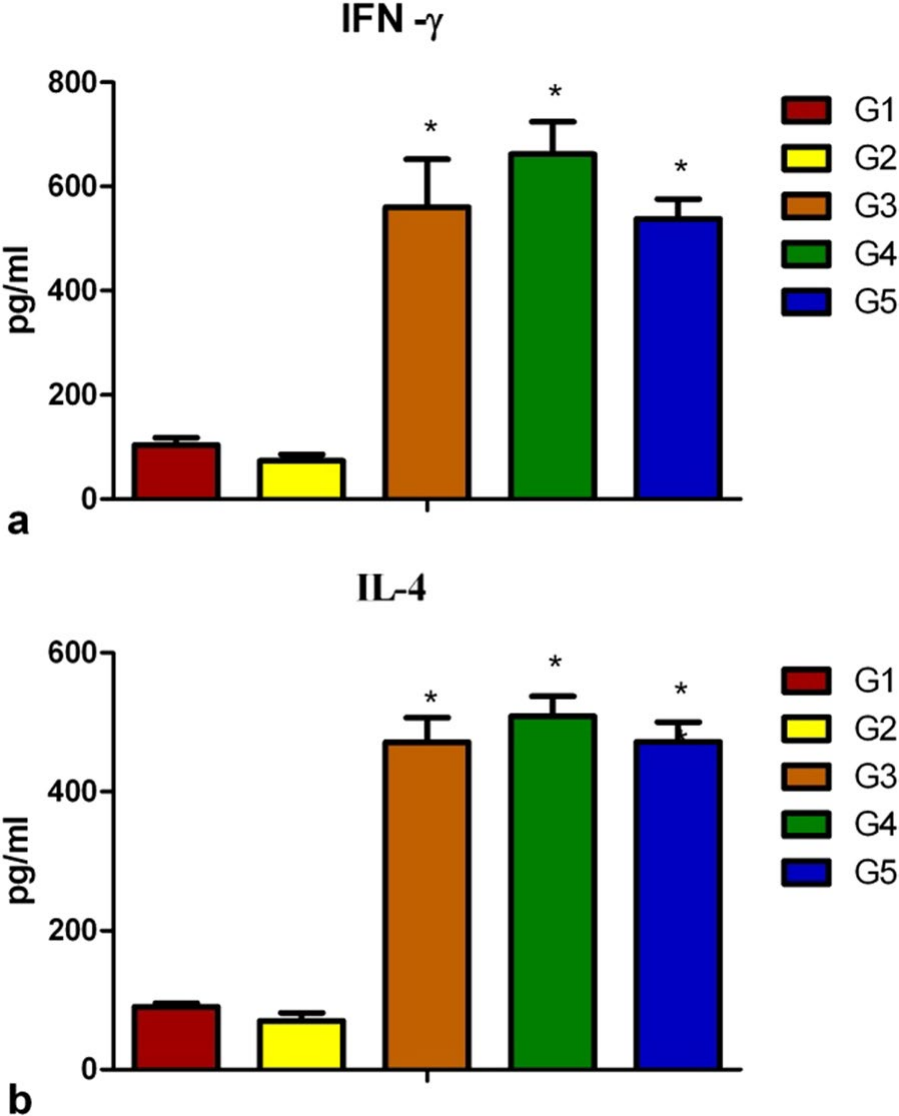
Cytokine production assays. Two weeks after the final immunization, three mice in each group were sacrificed and cells isolated from spleens then stimulated with soluble protein and VLPs for 40 h, cytokine production was quantified afterward. **a**, **b** represent the IFN-γ and IL-4 concentrations (pg/ml), respectively, in the supernatants from soluble protein and VLPs stimulated cells. Results are expressed as means ± standard deviations. *P < 0.05 were considered statistically significant

IFN-γ and IL-4 production in the spleen lymphocytes of the immunized mice were evaluated using a cytokine ELISA kit. In 4M2e-VLP, mHA-VLP, and cVLP group, the IFN-γ and IL-4 production levels in the spleen lymphocytes were significantly higher than that of the soluble group, as shown in Fig. 5. In the cVLP group, the IFN-γ and IL-4 production levels were 537 pg/ml and 471 pg/ml, respectively. In mHA-VLP group, the IFN-γ and IL-4 production levels were 662 pg/ml and 508 pg/ml, respectively. In 4M2e-VLP group, the production of IFN-γ and IL-4 levels were 560 pg/ml and 471 pg/ml, respectively. Compared to soluble protein group, the difference was statistically significant, and only background levels of cytokine-secreting cells were detected in the soluble protein group.

### VLPs protect mice from lethal virus challenge

**Fig. 6:**
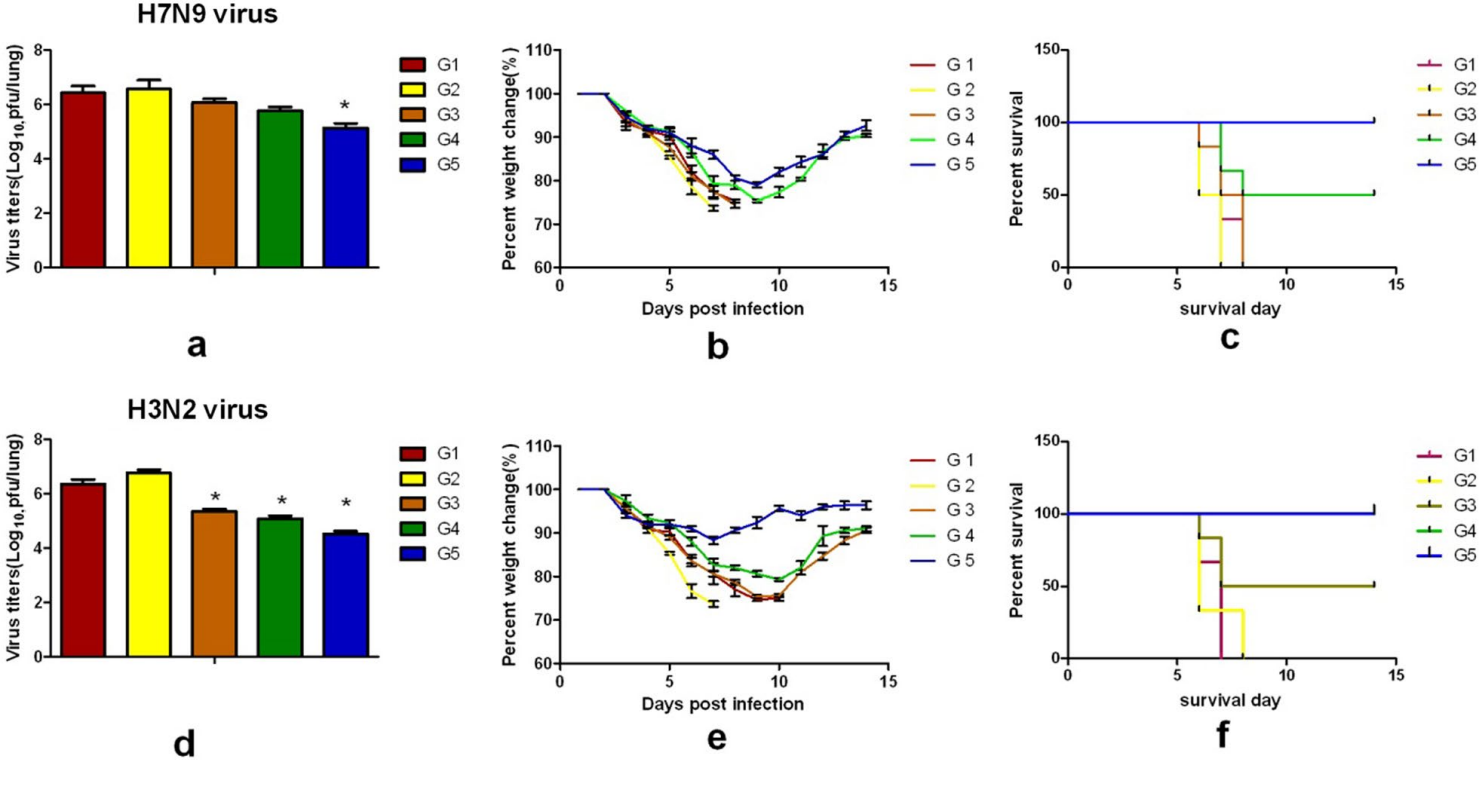
Protection efficacy against infection by H7N9 and H3N2 strains. The mice were challenged with lethal dose of virus in a volume 40 μl PBS through intranasal instillation. The body weight changes and survival rate were monitored daily for 14 days. The lung virus titers at day 4 post challenge were determined by standard plaque assays. Bars represent mean virus titers(Log_10_ pfu/ml) ± standard errors. **a**, **d**, lung virus titers. **b**, **e**, body weight changes. **c**, **f**, survival rate post challenge. *P < 0.05 were considered statistically significant: compared to G1 and G2 group

An effective influenza vaccine could limit the virus titers in the lungs post-challenge and conferred protection against viral infection. For the H7N9 (heterologous virus) challenge, as shown in Fig. 6, all the mice in the soluble protein immunized groups died 7–8d post- infection (p.i.) (Fig. 6c) with high titers of viruses in the lungs (Fig. 6a) and over 25% body weight loss (Fig. 6b). All mice in the 4M2e-VLP immunized group died with high titers of viruses in the lungs and over 20% body weight loss. Mice in the cVLP and mHA-VLP groups showed 100% and 50% survival rates, respectively, as well as effectiveness in reducing lung virus titers. cVLP effectively reduced virus titers from immunized mice compared to the soluble group with 1 × 10^5^ pfu/lung versus 2.5 × 10^6^ pfu/lung and 3.6 × 10^6^ pfu/lung after the H7N9 challenge. The mice immunized with cVLP had over 20-fold lower virus titers compared with those in the soluble group. Those in the mHA-VLP group showed an effective reduction of virus titers than those in the 4M2e-VLP group with 5 × 10^5^ pfu/lung versus 1 × 10^6^ pfu/lung. For the H3N2 challenge, the mice in the soluble 4M2e protein-immunized groups died 7–8 d p.i., and those in the soluble mHA protein-immunized group died 10 d p.i. (Fig. 6f). The cVLP and mHA-VLP immunization conferred full protection and significantly reduced the virus titers in the lungs (Fig. 6d). The mice in the mHA-VLP group showed 8%–10% bodyweight loss (Fig. 6e) and the 4M2e-VLP group had a 40% survival rate. The cVLP and mHA-VLP groups showed greater effectiveness in reducing virus titers from immunized mouse lungs post-challenge compared to the soluble group mice with 3.7 × 10^4^ pfu/lung and 1.1 × 10^5^ pfu/lung versus 2.1 × 10^6^ pfu/lung and 5.7 × 10^6^ pfu/lung after the H3N2 virus challenge. The mHA-VLP group showed a greater reduction in virus titers than the 4M2e-VLP group with 1.1 × 10^5^ pfu/lung versus 3.1 × 10^5^ pfu/lung. The mice that received three vaccinations with cVLP were fully protected and exhibited slight bodyweight loss after homologous or heterologous virus challenges. The mice in the mHA-VLP immunized group survived after the H3N2 challenge and showed partial protection after the H7N9 challenge, but exhibited some bodyweight loss. These results demonstrated that the immunization of mice with cVLP and mHA-VLP conferred more protection than those of the other groups after homologous or heterologous virus challenges and effectively reduced lung virus titers. Our findings also suggested that stem antibodies conferred better protection than M2e antibodies.

## Discussion

A universal influenza vaccine with a broadly cross-protective effect is a promising approach to the prevention of influenza infection. To contribute to research in this field, the present study mainly focused on conserved influenza virus proteins, including stalk domain of HA and M2e. As demonstrated, VLP particles had the advantage of native virus structure, improved antigen delivery to antigen-presenting cells (APCs), efficiently promoted APC activation [14, 18] and conferred protection or cross-protection against influenza viruses [19, 20].

In this study, stalk domain and 4M2e were directly incorporated into influenza VLPs, which induced high levels of humoral and mucosal immune responses in mice. Higher IgG titers were obtained from the VLP-immunized group compared to those from the soluble protein group. In the cVLP group, the stalk domain stimulates higher levels of antibodies than 4M2e. Antigen competition by much more stalk domain-specific B cells for a limited amount of immunogenic entities may suppress the 4M2e specific antibody responses. More stalk domain-specific precursor B cells were expected to be captured, leaving only a few for stimulation of the few 4M2e specific precursor B cells. The same phenomenon results in a suppression of the NA-specific antibody response by immunodominant HA-specific B cells [21]. In 4M2e-VLP, 4M2e were directly incorporated to VLP particles in the absence of HA and NA, 4M2e could be delivered to immune cells at a high epitope density to overcome the limitations of 4M2e presentation during viral infection or vaccination, thus, such competition can be avoided by presenting individual antigens on physically distinct immunogenic entities to the immune system. In this study, the stalk domain and 4M2e were incorporated into influenza virus particles, the stalk domain and 4M2e could be delivered to the immune cells at a high epitope density and presented on the nanocluster surface, mimicking natural presentation, even at low concentrations, the stalk domain-VLPs and 4M2e-VLPs can effectively induce high levels of specific antibodies. It is established that the VLPs are similar to the infectious virus and can effectively induce innate and adaptive immune responses, as well as increase the immunogenicity of weak antigens [22].

Our findings showed that VLPs also induced mucosal IgA responses. The IgA titer in nasal washes were improved. In the mucosal system(the first barrier against pathogens), the IgA antibody secreted in the respiratory tract plays an important role in the defense against pathogens [23]. Our data demonstrated that the stalk domain/4M2e-VLP stimulated the mucosal antibody in mice. These mucosal antibodies may contribute to protective immunity. Cellular immune responses are important in generating and regulating an effective immune response and are known to contribute to broad cross-protective immunity. We believe that cellular immune responses also contribute to protection against experimental infection in mice.

Results of our study suggest that the cVLP and mHA-VLP groups generated better protection than the 4M2e-VLP group, as demonstrated by body weight loss, lung virus titers, and survival rate after the challenge. These stalk domain-specific antibodies probably inhibited the critical step of the virus host membrane fusion during the influenza virus entry. The stem antibodies also interfered with the release of progeny viruses. Antibody-dependent cell-mediated cytotoxicity (ADCC) activity are potentially important mechanisms in protective immunity. Previous work reported that ADCC plays a role in protection against influenza, and suggested that HA stem antibodies were more effective at inducing ADCC [24, 25]. Our studies demonstrated that VLPs group elicited higher ADCC activity and induced cross-reactive ADCC antibodies to H7N9, and this may be one of the mechanisms by which antibodies mediate protection in our challenge model. The 4M2e specific antibody had no neutralizing effect [26], and its protective effect may depended on ADCC, opsonization, and complement activation [13]. Opsonization by anti-M2e IgG contributed to in vivo immune protection, but was less effective given the low abundance of M2 molecules compared with HA and NA on influenza viral particles. M2 protein was expressed at least as abundantly as NA on the surface of the infected cells, but much less than M2 protein (with an ectodomain of 23 amino acids) and molecules were incorporated into the virion compared with HA or NA [27]. Many studies had indicated that M2-specific antibody responses were poorly induced in human influenza infection and, if induced, appeared to be of low titer and short duration. The mature protein M2 were displayed at high density (approximately 50% of density of HA in infected cells during the stage of virus maturation) but at low density (1–2% of HA) in the membrane of mature virus particles. The low density of M2 displayed on the virus particles could suppress the protection mediated by M2-specific Ab.

M2e was conserved in influenza A viruses. Variations of M2e sequences in different virus strains also limited the protective efficacy of M2e vaccines against viral infections. 4M2e-VLP conferred better protection in mice with limited body weight loss after the H3N2 challenge. The M2e sequence of H3N2 was identical to that of the 4M2e-VLP used for immunization in this study. In contrast, although survived, the mice challenged by H7N9 showed much more severe illness as demonstrated by increased body weight loss and high lung virus titers. This may be a result of 6 amino acid substitutions in the M2e of H7N9 (M S L L T EVETP**T**R**TG**W**E**C**N**C**S**G). The difference of the stalk domain between H3N2 and H7N9 may also contribute to different protection results in this study.

## Conclusions

Our research found that immune responses against the stalk domain showed better effectiveness in reducing disease symptoms in immunized mice upon lethal viral challenge than M2e. Other factors such as mucosal immunity and T-cell responses may have also contributed to the protection. The particle formulations described in this study promoted immunological advantages of particulate antigens and conferred cross-protection in mice. Our study demonstrated that the assembled VLPs were promising as a potential universal influenza vaccine.

## Data Availability

The datasets used and/or analysed during the current study are available from the corresponding author on reasonable request.

## References

[1] World Health Organization. Influenza (Seasonal). World Health Organization (2018).

[2] Gething MJ, Bye J, Skehel J, Waterfield M (1980). Cloning and DNA sequence of double-stranded copies of haemagglutinin genes from H2 and H3 strains elucidates antigenic shift and drift in human influenza virus. Nature.

[3] Jou WM, Verhoeyen M, Devos R, Saman E, Fang R, Huylebroeck D, Fiers W, Threlfall G, Barber C, Carey N, Emtage S (1980). Complete structure of the hemagglutinin gene from the human influenza A/Victoria/3/75 (H3N2) strain as determined from cloned DNA. Cell.

[4] Klimov A, Simonsen L, Fukuda K, Cox N (1999). Surveillance and impact of influenza in the United States. Vaccine.

[5] Bridges CB, Thompson WW, Meltzer MI, Reeve GR, Talamonti WJ, Cox NJ, Lilac HA, Hall H, Klimov A, Fukuda K (2000). Effectiveness and cost-benefit of influenza vaccination of healthy working adults: a randomized controlled trial. JAMA.

[6] Gerdil C (2003). The annual production cycle for influenza vaccine. Vaccine.

[7] Xavier S (2019). The role of matrix protein 2 ectodomain in the development of universal influenza vaccines. J Infect Dis.

[8] Fiers W, De Filette M, Birkett A, Neirynck S, Min JW (2004). A “universal” human influenza A vaccine. Virus Res.

[9] Krystal M, Elliott RM, Benz EJ, Young JF, Palese P (1982). Evolution of influenza A and B viruses: conservation of structural features in the hemagglutinin genes. Proc Natl AcadSci USA.

[10] Steel J, Lowen AC, Wang TT (2010). Influenza virus vaccine based on the conserved hemagglutinin stalk domain. MBio.

[11] Krammer F, Pica N, Hai R, Margine I, Palese P (2013). Chimeric hemagglutinin influenza virus vaccine constructs elicit broadly protective stalk-specific antibodies. J Virol.

[12] Neirynck S, Deroo T, Saelens X (1999). A universal influenza A vaccine based on the extracellular domain of the M2 protein. Nat Med.

[13] El Bakkouri K, Descamps F, De Filette M (2011). Universal vaccine based on ectodomain of matrix protein 2 of influenza A: Fc receptors and alveolar macrophages mediate protection. J Immunol.

[14] Gao DD, Chen Y, Han D (2017). Membrane-anchored stalk domain of influenza HA enhanced immune responses in mice. Microb Pathog.

[15] Wang BZ, Xu R, Quan FS, Kang SM, Wang L, Compans RW (2010). Intranasal immunization with influenza VLPs incorporating membrane-anchored flagellin induces strong heterosubtypic protection. PLoS ONE.

[16] Cox F, Baart M, Huizingh J, Tolboom J, Dekking L, Goudsmit J (2015). Protection against H5N1 influenza virus induced by matrix-m adjuvanted seasonal virosomal vaccine in mice requires both antibodies and T cells. PLoS ONE.

[17] Co MD, Terajima M, Thomas SJ, Jarman RG, Rungrojcharoenkit K, Fernandez S (2014). Relationship of preexisting influenza hemagglutinationinhibition, complement-dependent lytic, and antibody-dependent cellular cytotoxicity antibodiesto the development of clinical illness in a prospectivestudy of A(H1N1)pdm09 influenza in children. Viral Immunol.

[18] Mohsen MO, Zha L, Cabral-Miranda G, Bachmann MF (2017). Major findings and recent advances in virus-like particle (VLP)-based vaccines. Semin Immunol.

[19] Wang BZ, Gill HS, He C (2014). Microneedle delivery of an M2e-TLR5 ligand fusion protein to skin confers broadly cross-protective influenza immunity. J Control Release.

[20] Wang L, Chang TZ, He Y, Kim JR, Wang S (2017). Coated protein nanoclusters from influenza H7N9 HA are highly immunogenic and induce robust protective immunity. Nanomedicine.

[21] Hartmann G, Weeratna RD, Ballas ZK, Payette P, Blackwell S, Suparto I, Rasmussen WL, Waldschmidt M, Sajuthi D, Purcell RH, Davis HL, Krieg AM (2000). Delineation of a CpG phosphorothioate oligodeoxynucleotide for activating primate immune responses in vitro and in vivo. J Immunol.

[22] Gao Y, Wijewardhana C, Mann JFS (2018). Virus-like particle, liposome, and polymeric particle based vaccines against HIV-1. Front Immunol.

[23] Asahi Y, Yoshikawa T, Watanabe I, Iwasaki T, Hasegawa H, Sato Y (2002). Protection against influenza virus infection in polymeric Ig receptor knockout mice immunized intranasally with adjuvant-combined vaccines. J Immunol.

[24] Laidlaw BJ, Decman V, Ali MA, Abt MC, Wolf AI, Monticelli LA (2013). Cooperativity between CD8+ T cells, non-neutralizing antibodies, and alveolar macrophages is important for heterosubtypic influenza virus immunity. PLoS Pathog.

[25] De Jong NMC, Aartse A, Van Gils MJ, Eggink D (2020). Development of broadly reactive influenza vaccines by targeting the conserved regions of the hemagglutinin stem and head domains. Expert Rev Vaccines.

[26] Schotsaert M, De Filette M, Fiers W, Saelens X (2009). Universal M2 ectodomain-based influenza A vaccines: preclinical and clinical developments. Expert Rev Vaccines.

[27] Zebedee SL, Lamb RA (1988). Influenza A virus M2 protein: monoclonal antibody restriction of virus growth and detection of M2 in virions. J Virol.

